# Integrating multi-omics techniques and *in vitro* experiments reveals that GLRX3 regulates the immune microenvironment and promotes hepatocellular carcinoma cell proliferation and invasion through iron metabolism pathways

**DOI:** 10.3389/fimmu.2024.1496886

**Published:** 2024-11-25

**Authors:** Yang Li, Yuan Chen, Yang Zhang, Yunsheng Fang, Ling Wu, Ying Zhao, Danqiong Wang, Xiaoyuan Qiao

**Affiliations:** ^1^ Department of General Medicine, Shanxi Bethune Hospital, Shanxi Academy of Medical Sciences, Third Hospital of Shanxi Medical University, Tongji Shanxi Hospital, Taiyuan, China; ^2^ Department of Geriatrics, Tongji Hospital, Tongji Medical College, Huazhong University of Science and Technology, Wuhan, China; ^3^ School of Mechanical Engineering, Taiyuan University of Science and Technology, Taiyuan, China; ^4^ The Key Laboratory of Biomedical Information Engineering of Ministry of Education, School of Life Science and Technology, Xi’an Jiaotong University, Xi’an, China; ^5^ Bioinspired Engineering & Biomechanics Center (BEBC), Xi’an Jiaotong University, Xi’an, China; ^6^ Tumor Center, Shanxi Bethune Hospital, Shanxi Academy of Medical Sciences, Third Hospital of Shanxi Medical University, Tongji Shanxi Hospital, Taiyuan, China; ^7^ Department of Comprehensive Medicine, Shanxi Province Cancer Hospital/Shanxi Hospital Affiliated to Cancer Hospital, Chinese Academy of Medical Sciences/Cancer Hospital Affiliated to Shanxi Medical University, Taiyuan, China

**Keywords:** iron metabolism, GLRX3, immunotherapy, precision medicine, multi-omics analysis, immune signatures, personalized therapy

## Abstract

**Background:**

Hepatocellular carcinoma (HCC) is a common malignancy worldwide, and its development is closely related to abnormalities in iron metabolism. This study aims to systematically analyze changes in iron metabolism in the tumor microenvironment of HCC using single-cell sequencing technology, and investigate the potential mechanisms by which iron metabolism regulation affects the survival of liver cancer patients.

**Materials and methods:**

Single-cell sequencing data from hepatocellular carcinoma patients were obtained from the GEO database. By iron metabolism genomic scoring, we assessed differences in iron metabolism levels in hepatocellular carcinoma samples. By cell communication analysis as well as GO and KEGG enrichment analysis, we determined the functional role of iron metabolism in different cell types. We used survival analysis and Kaplan-Meier curves to assess the impact of iron metabolism levels on patient prognosis. In addition, we identified and analyzed the expression profile of the GLRX3 gene, investigated its key regulatory role in iron metabolism, and validated its clinical value as a prognostic marker. Finally, we explored the effect of GLRX3 on hepatocellular carcinoma phenotype by *in vitro* experiments such as PCR, transwell, CCK8, and wound healing assay.

**Results:**

Bioinformatics results and experimental validation confirmed the dysregulation of iron metabolism in the development of hepatocellular carcinoma, revealing iron’s regulatory influence across various cell types. Additionally, GLRX3 was identified as a key regulatory factor in iron metabolism, and the mechanism by which GLRX3 regulates tumor cell proliferation and immune evasion was determined. Furthermore, experiments verified GLRX3’s role in facilitating tumor cell proliferation and invasion.

**Conclusion:**

This study highlights the critical role of iron metabolism in the progression of hepatocellular carcinoma, particularly the regulatory mechanism of the GLRX3 gene in tumor cell proliferation and immune evasion. Iron metabolism abnormalities are not only drivers of liver cancer development but also key indicators of patient prognosis.

## 1 Introduction

Primary liver cancer (PLC) is the fifth most common cancer globally and the second leading cause of cancer-related deaths (1–4). Its incidence and mortality rates are rising rapidly, particularly in Western countries. Hepatocellular carcinoma (HCC), the most prevalent form of PLC, makes up 90% of all primary liver tumors and around 5% of all cancers (5–8). The malignant transformation of hepatocytes results in HCC (9), with known risk factors including excessive alcohol consumption, hepatitis B virus (HBV) infection, fat accumulation in the liver, and autoimmune liver diseases (10–12). While liver transplantation, surgery, and local therapies can be curative at early stages (13, 14), most liver cancer patients are diagnosed late, where treatment options are extremely limited and the prognosis is poor (15–17). Thus, understanding changes in the tumor microenvironment during liver cancer progression and gaining deeper insights into its pathogenesis are critical for developing effective treatments (18).

Iron metabolism encompasses the comprehensive processes of iron absorption, transport, storage, and utilization within a biological system. Although the body’s requirement for iron is relatively modest, it is an essential trace element that plays a critical role in numerous physiological processes (19). The key components involved in maintaining cellular iron homeostasis include transferrin receptor 1 (TfR1), which internalizes transferrin-bound iron; ferroportin (Fpn), the sole iron export protein; and ferritin, which stores excess iron (20). Iron levels are tightly regulated at both systemic and cellular levels to remain within an optimal range. However, excessive iron can promote the production of highly reactive and toxic oxidants via the Fenton reaction, impairing immune function and disrupting various physiological processes (21).

Hepatocytes play a crucial role in maintaining stable plasma glucose and lipoprotein levels in humans (22). Under normal conditions, hepatocytes remain quiescent; however, when liver tissue is excessively exposed to viruses, toxic substances, or metabolites, significant physiological changes occur. Given that the liver is a primary organ for excess iron accumulation, it plays a crucial role in maintaining iron homeostasis (19, 23). Dysregulation of iron metabolism significantly increases the risk of liver cancer. Research has shown that iron overload is not only associated with cancer development but also actively contributes to carcinogenesis. Excess iron induces oxidative stress-mediated DNA damage in hepatocytes and promotes the rapid proliferation of tumor cells (24, 25). Thus, understanding the intrinsic link between abnormal iron metabolism and changes in the tumor microenvironment of liver cancer is critical for developing precise treatment strategies and for uncovering the broader impact of metal ions on cancer progression (22, 26).

## 2 Materials and methods

### 2.1 Cell culture

The human hepatocellular carcinoma cell lines, Hep3B and Huh7, were cultured in RPMI/1640 medium (Gibco) supplemented with 10% fetal bovine serum (FBS) (Hyclone), along with 100 U/L of penicillin and 100 mg/L of streptomycin (Thermo Fisher). Cells were maintained at 37°C in a humidified atmosphere of 5% CO2. The culture medium was changed every 2-3 days to ensure optimal growth conditions. When cells reached 80-90% confluence, they were passaged using trypsin-EDTA for further experiments.

### 2.2 shRNA knockdown

Plasmids expressing shRNA, specifically designed to target GLRX3, were carefully constructed with the assistance of GenePharma. During cultivation, the cells were treated with viral supernatants and polybrene (Sigma Aldrich) in the culture medium. After 24 hours of incubation, the cells were transferred to fresh medium containing 2.0 μg/ml of puromycin. The efficiency of GLRX3 knockdown was confirmed two days later using qRT-PCR analysis.

### 2.3 qPCR assay

Total RNA extraction was carried out utilizing the RNA Eazy Fast Tissue/Cell Kit (TIANGEN Biotech) in accordance with the manufacturer’s guidelines. Subsequently, cDNA synthesis was performed using the FastKing RT Kit (TIANGEN Biotech), adhering to the provided protocol. Real-time PCR analysis was conducted with the application of the SuperReal PreMix Plus (TIANGEN Biotech) reagent, implemented on the StepOnePlus Real-Time PCR System. The PCR reaction encompassed an initial pre-denaturation phase at 95°C for 15 minutes, followed by 40 amplification cycles, comprising denaturation at 95°C for 10 seconds, annealing at 72°C for 20 seconds, and extension at 60°C for 20 seconds. Primer sequences utilized were procured from Sangon Biotech. (Species of Human Origin) GLRX3 Forward Primer: GGGCGGCTGAGGCAGCT,reverse primer GCAGGGGGCAGCATGAGTC;(Species of Human Origin) IL10 Forward Primer: GACTTTAAGGGTTACCTG GGTTG,Reverse Primer: TCACATGCGCCTTGATGTCTG; At last, PCR signals 2-44Ct was used to calculate the expression of genes mRNA levels. The following sequences were used: 5′-GTGGAAATTCTTCACAAACAT-3′ for human GLRX3 shRNA and 5′-GGAATCTCATTCGATGCATAC-3′ for the control shRNA.

### 2.4 Transwell assay

A seeding density of 1×10^5 cells was allocated to either Matrigel-coated chambers (BD Biosciences, San Jose, CA) for the invasion assay or uncoated chambers designated for the migration assay. The upper chamber was filled with serum-free medium, while the lower chamber was supplied with complete RPMI/1640 medium. Following a 24-hour incubation period, cells that had traversed the membrane were meticulously fixed with a 4% paraformaldehyde solution and subsequently subjected to staining with 0.1% crystal violet. Cell quantification was carried out using a light microscope, specifically the Thermo Fisher instrument based in Waltham, MA, USA.

### 2.5 CCK-8 assay

Cell viability was ascertained via the Cell Counting Kit-8 (CCK-8) assay. Cells were appropriately seeded at a density of 1500 cells per well, and each well contained 200 µl of complete medium within 96-well plates. Subsequent to seeding, the cells were diligently cultured under standard conditions at 37°C. Following each experimental procedure, 20 µl of CCK-8 reagent (Beyotime) was introduced into every well. A further incubation period of 2 hours ensued, after which the optical density value (OD450nm) was meticulously determined utilizing a microplate reader.

### 2.6 Wound healing assay

A wound healing assay was conducted to evaluate the migratory capacity of hepatocellular carcinoma cells. Transfected cells in six-well plates were incubated at 37°C until they reached around 80% confluence. Then, a 200 μL sterile pipette tip was used to create uniform wounds in the cell monolayer. Cells were washed twice with phosphate-buffered saline to remove any debris, and the medium was replaced with serum-free medium. Cell migration into the wound area was carefully monitored under an Olympus inverted microscope at 0 and 24 hours.

### 2.7 Protein expression and immunohistochemistry

We used the CTPAC database to validate the difference in the expression of GLRX3 protein in hepatocellular carcinoma tissues and normal liver tissues. The expression levels of GLRX3 in hepatocellular carcinoma tissues and normal tissues were verified by immunohistochemical sections from the HPA database.

### 2.8 Data sources

The single-cell sequencing data used in this study was obtained from the GEO database, specifically from dataset GSE149614, which includes sequencing data from 10 HCC patients. We selected two types of samples, primary tumors and non-tumorous liver tissues, for analysis. Spatial transcriptomics sequencing data from one HCC tumor tissue sample was sourced from GSM6177612, with tissue sections derived from primary hepatocellular carcinoma regions. Additionally, RNA-seq data for HCC was downloaded from the UCSC Xena platform (https://xena.ucsc.edu/), originating from the TCGA (The Cancer Genome Atlas) cohort. This dataset contains sequencing information from 424 samples along with corresponding survival data, which was used for survival analysis. External validation sets utilized in this study included GSE144269, GSE76427, and ICGC_LIRI.

### 2.9 Single-cell sequencing data processing

After processing single-cell sequencing data from 10 tumor and 8 normal liver samples, we obtained a total of 63,101 cells. Preliminary data analysis was conducted using the Seurat package, which included quality control, dimensionality reduction, clustering, and visualization. To ensure the reliability of the sequencing data, stringent quality control measures were implemented. Specifically, cells with fewer than 500 or more than 6,000 detected genes, as well as those with over 20% mitochondrial gene content, were excluded. This step minimized the presence of empty droplets, doublets, and senescent cells. Following data normalization and scaling, PCA-based dimensionality reduction was performed, and batch effects were mitigated using the Harmony package. We then selected the top 20 principal components for clustering with a resolution of 0.3, resulting in the identification of 17 cell clusters, which were visualized using UMAP (27–29).

### 2.10 Cell type identification and subpopulation segmentation

We employed common cell marker genes and the “FindAllMarkers” function to conduct preliminary cell type identification. Based on the expression patterns of marker genes in each cluster and the upregulation of specific genes, we assigned cell type labels. Subpopulations within larger groups, such as myeloid cells, B cells, and T/NK cells, were further subdivided. Using a resolution of 0.1, we identified distinct cell types, including plasma cells, cytotoxic T lymphocytes (CTLs), epithelial-mesenchymal transition cells (EMTs), regulatory T cells (Tregs), and macrophages (30, 31).

### 2.11 Tumor cell identification and stemness assessment

To identify tumor cells, we utilized the “copykat” package for copy number variation (CNV) analysis. CopyKAT (Copy-number Karyotyping of Tumors) is a computational tool that employs an integrative Bayesian approach to detect whole-genome aneuploidy in single cells at a 5MB resolution, allowing us to distinguish tumor cells from normal cells. Cells displaying extensive whole-genome CNV (aneuploidy) were classified as tumor cells, while stromal and immune cells typically exhibited 2N diploid or near-diploid CNV profiles. To assess the differentiation status of tumor cells and support pseudotime analysis of T cell subpopulations, we applied the “cytotrace” package for cell stemness scoring. Cytotrace provides a continuous measure of developmental potential, ranging from 0 (fully differentiated) to 1 (pluripotent). Pseudotime inference for T cell subpopulations was performed using the “monocle” package (32).

### 2.12 Iron metabolism level assessment

To quantify iron metabolism levels across different cell types using 73 iron metabolism-related genes, we applied several gene set scoring methods, including AddModuleScore, ssGSEA, AUCell, UCell, and singscore. Each method generated a score for each cell, and after centering and standardizing these scores, the final score for each cell was obtained by summing the five scores. The use of multiple scoring methods helps reduce errors and biases in gene set scoring, providing more comprehensive information, increased robustness, and better biological interpretation. For cell types that showed significant changes in iron metabolism levels between groups, cells were classified into high- and low-score groups based on the average score, representing different levels of iron metabolism (27, 33, 34).

### 2.13 Enrichment and cell communication analysis

To investigate the biological functional differences among cells with varying iron metabolism levels, we conducted Gene Ontology (GO) and Kyoto Encyclopedia of Genes and Genomes (KEGG) enrichment analyses. Genes for enrichment analysis were identified using the “FindMarkers” function, focusing on those upregulated in the high-score group cells. The “clusterProfiler” package was employed to retrieve gene sets from the GO, KEGG, and GSEA databases and to visualize the results. Additionally, we utilized the “GSVA” package, which employs the “HALLMARK” gene set to identify tumor-associated biological processes. To compare differences in cell communication between high- and low-score cells, we conducted cell communication network analysis using the “CellChat” package. CellChat simulates and analyzes intercellular communication by integrating gene expression data with known interactions between signaling ligands, receptors, and cofactors (35–37).

### 2.14 Infiltration and prognostic analysis of high- and low-score cells

Using the marker genes of high- and low-score cells, we performed ssGSEA scoring on TCGA data to classify patients into high- and low-infiltration groups. Survival data from these groups were then used to plot Kaplan-Meier (K-M) curves, allowing us to compare prognostic differences. The “survival” and “survminer” packages were employed to plot K-M curves for both overall survival and progression-free survival (38).

### 2.15 Spatial transcriptomics data deconvolution analysis

For the initial processing of spatial transcriptomics data, we utilized the “Seurat” package. During quality control, only mitochondrial and ribosomal genes were excluded, while data for each spot were retained. After normalization and centering using the “SCTransform” function, we performed PCA-based dimensionality reduction and clustering. We clustered the data using the top 20 principal components, resulting in the identification of 7 cell clusters. The “scMetabolism” package was employed to infer metabolic activity in each cell cluster from the spatial transcriptomics data. This package includes human-specific metabolic gene sets covering 85 KEGG pathways and 82 REACTOME entries, employing the VISION algorithm to score each cell. To address the resolution limitations of spatial transcriptomics and leverage spatial location information, deconvolution analysis was performed using the “spacexr” package, specifically the RCTD deconvolution analysis. Annotated single-cell data were used to deconvolute spatial transcriptomics data, inferring the probability of each cell type at each sequencing spot. Cells from high- and low-score groups were also included in the analysis to compare iron metabolism levels across different locations.

### 2.16 Expression and prognostic analysis of key iron metabolism genes in tumors

For key iron metabolism genes, we performed differential gene expression analysis using TCGA data and validated the results with GEO data. Six significantly differentially expressed iron metabolism genes were then used to score bulk data, categorizing patients into high and low groups for comparison of prognostic differences, reflecting the impact of key iron metabolism genes on HCC prognosis. The ssGSEA method was employed to score and plot K-M curves using various survival datasets. Two additional datasets from GEO and ICGC were used as external validation sets to assess the impact of key iron metabolism genes on HCC patient prognosis. Furthermore, we examined the expression of key iron metabolism genes in spatial transcriptomics data, comparing gene expression in normal cells, mixed cells, and malignant cells, and their correlation with various cell types.

### 2.17 Prognostic and clinical analysis of GLRX3, a key iron metabolism gene

For GLRX3, a key gene in iron metabolism, we conducted subgroup differential expression analysis using clinical information from TCGA. The prognostic value of GLRX3 was evaluated using TCGA and multiple external validation sets. Enrichment analysis and spatial transcriptomics data were also employed in the study of GLRX3.

### 2.18 Statistical analysis

Statistical analyses were performed using R 4.2.2 64-bit version and its supported packages. The non-parametric Wilcoxon rank-sum test was used to assess relationships between groups for continuous variables. Spearman correlation analysis was used to test correlation coefficients. All statistical analyses were considered significant at P<0.05.

## 3 Results

### 3.1 Quality control of liver cancer samples

**Figure 1:**
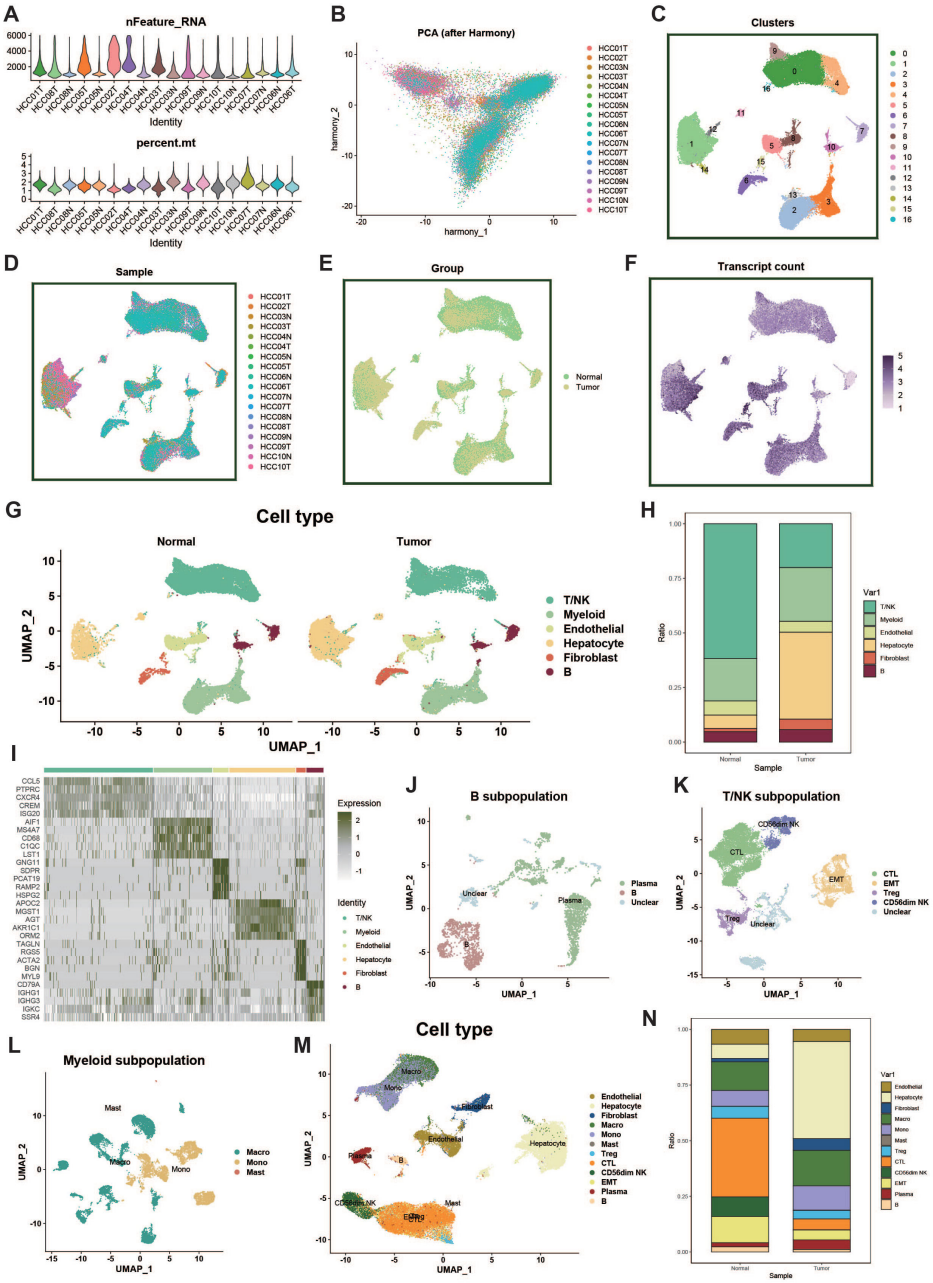
Single-cell data processing and cell type identification. **(A)** Violin plot showing sample characteristics after single-cell data quality control. The upper plot displays the number of detected genes, while the lower plot shows the proportion of mitochondrial genes. **(B)** PCA plot of cell distribution across samples after batch effect removal. **(C)** UMAP of dimensionality-reduced clustered cell populations, with a total of 17 clusters. **(D)** UMAP showing cell distribution across different samples. **(E)** UMAP showing cell distribution across different groups. **(F)** UMAP of cell counts. **(G)** Results of cell type identification, displaying the distribution and number of each cell type in different groups. **(H)** Bar plot of cell proportions. **(I)** Heatmap of cell marker gene expression. **(J)** UMAP of B cell subpopulations. **(K)** UMAP of T/NK cell subpopulations. **(L)** UMAP of myeloid cell subpopulations. **(M)** UMAP of overall cell types. **(N)** Bar plot showing proportions of overall cell types.

In this study, we obtained single-cell transcriptomic data from the GEO database (dataset GSE149614), which includes 18 liver cancer tumor tissue samples and adjacent normal liver tissue samples from ten patients. To ensure high-quality single-cell data analysis, we first performed quality control on all samples. To minimize the impact of aging cells, red blood cells, and a high percentage of mitochondrial reads, we evaluated key quality metrics, such as UMI counts and the percentages of mitochondrial and hemoglobin gene expression (**Figure 1A**). Additionally, we employed the Harmony package to correct for potential batch effects in sequencing, ensuring that observed differences were due to biological variation between samples (**Figure 1B**). After dimensionality reduction and clustering, we visualized 61,776 cells that passed quality control, which were grouped into 16 distinct clusters via UMAP (**Figure 1C**). Furthermore, we analyzed differences in data distribution across samples (**Figure 1D**), between tumor and normal groups (**Figure 1E**), and in mRNA density (**Figure 1F**).

Next, using common cell marker genes and the “FindAllMarkers” function, we performed preliminary cell type identification. Based on the expression patterns of marker genes and upregulated genes, we named the cell types (**Figure 1G**). **Figure 1H** displays the distribution of different cell types across the tumor and normal groups, while the heatmap in **Figure 1I** shows the marker genes for each cell cluster. For the large groups of myeloid cells, B cells, and T/NK cells, we further subdivided the populations, using a resolution of 0.1, identifying plasma cells, CTLs, EMTs, Tregs, macrophages, and more (**Figures 1J–M**). Lastly, we displayed the distribution differences of all cell types across the tumor and normal groups (**Figure 1N**).

### 3.2 Tumor cell identification

**Figure 2:**
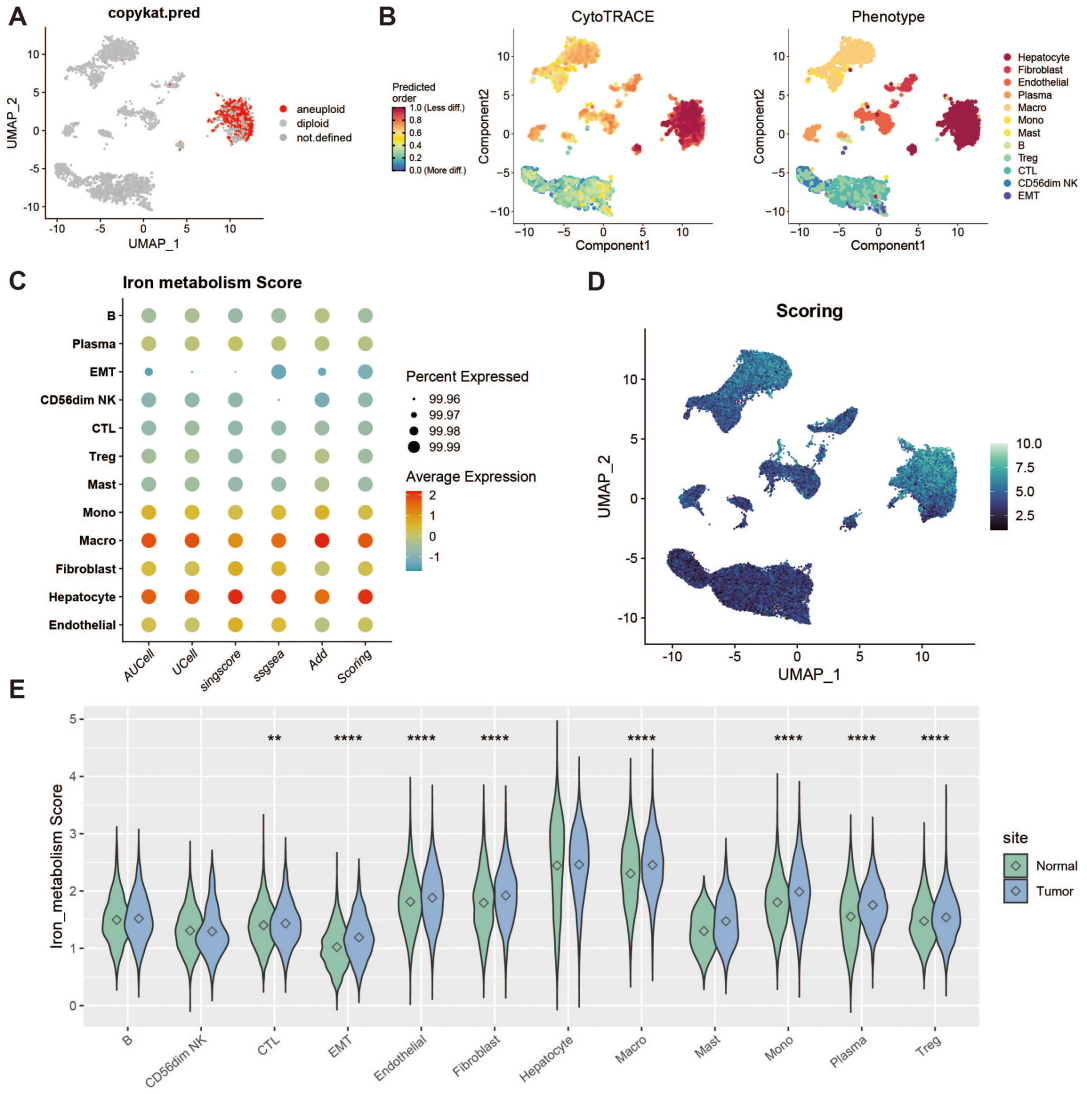
Tumor microenvironment analysis and iron metabolism level assessment. **(A)** UMAP showing copy number variation, where red indicates polyploid cells (tumor cells). **(B)** Heatmap of stemness score, ranging from 0 (differentiated) to 1 (pluripotent). **(C)** Bubble plot of gene set scoring results. **(D)** Heatmap of gene set scores. **(E)** Violin plot of score differences between tumor and normal groups. ** represents a p-value < 0.01, **** represents a p-value < 0.0001.

To identify tumor cells, we used the “copykat” package for copy number variation (CNV) analysis, which distinguishes tumor from normal cells by identifying aneuploidy. Cells exhibiting extensive genome-wide CNV were classified as tumor cells. **Figure 2A** shows a group of cells with high levels of CNV abnormalities detected by copykat. Additionally, we performed stemness scoring using the “cytotrace” package (**Figure 2B**). By combining these results with those from copykat, we confirmed that hepatocytes constituted a highly malignant tumor cell population. Next, we applied five scoring methods (AddModuleScore, ssGSEA, AUCell, UCell, and singscore) to assess the expression of iron metabolism genes across different cell populations (**Figures 2C, D**). We also compared the iron metabolism scores of each cell type between tumor and normal groups (**Figure 2E**).

### 3.3 Plasma cell iron metabolism analysis

**Figure 3:**
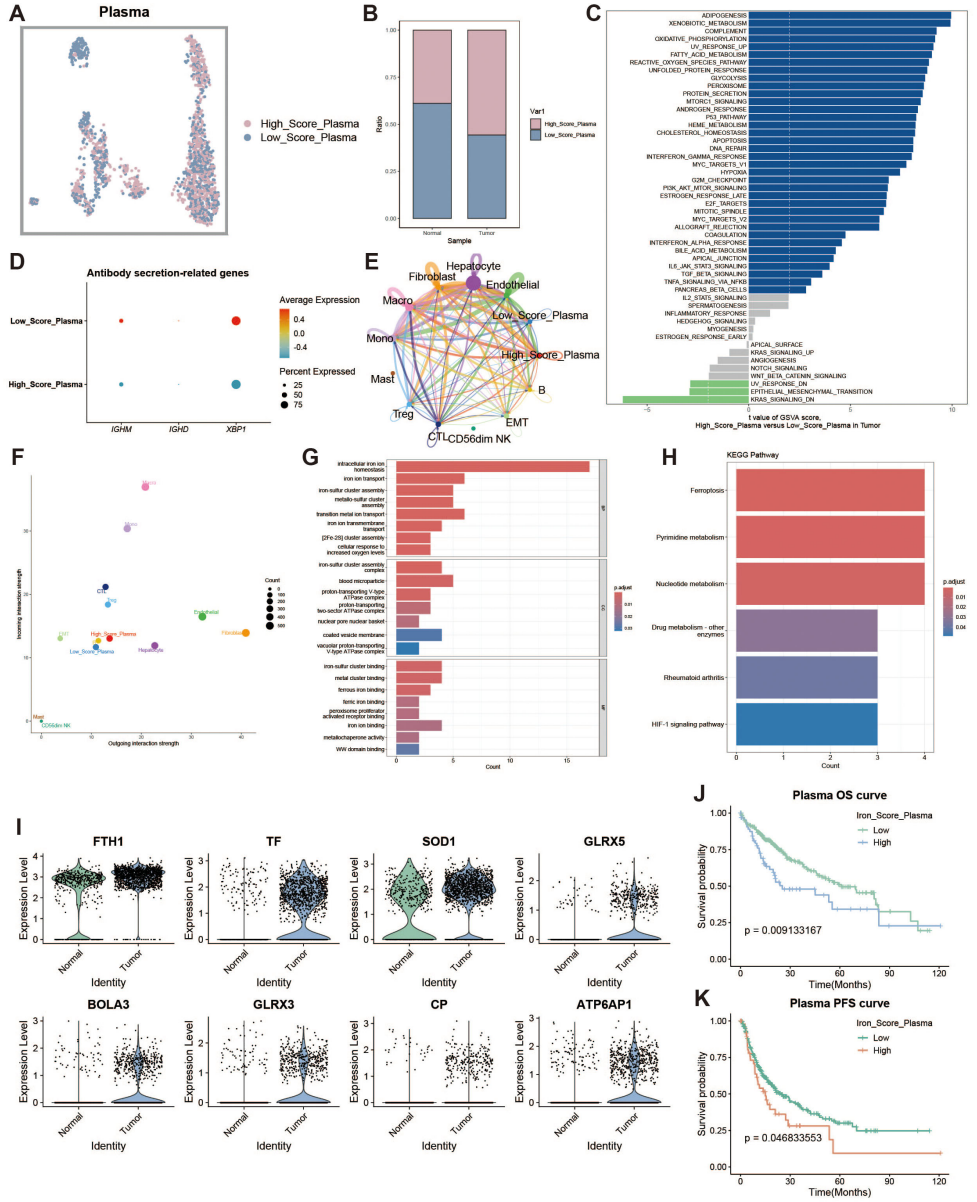
Plasma cell analysis. **(A)** UMAP of high and low iron metabolism score cells, where pink indicates high iron metabolism score cells and blue indicates low score cells. **(B)** Bar plot of cell proportions. **(C)** Diverging bar plot of GSVA enrichment results for Hallmark gene sets, showing pathways enriched in low-score and high-score cells. **(D)** Bubble plot of antibody secretion-related gene expression. **(E)** Circle plot showing cell communication frequency, where line thickness represents the number of communications. **(F)** Scatter plot of signal transmission and reception strength between cells. **(G)** Bar plot of GO enrichment analysis results. **(H)** Bar plot of KEGG enrichment analysis results. **(I)** Violin plot of differential expression of iron metabolism-related genes in plasma cells. **(J)** KM survival curve for high and low infiltration groups (overall survival). **(K)** KM survival curve for high and low infiltration groups (progression-free survival).

To investigate the characteristics of iron metabolism in plasma cells within the liver cancer tumor microenvironment, we categorized plasma cells into high and low expression groups based on their iron metabolism scores (**Figure 3A**). Notably, plasma cells in the tumor group exhibited significantly higher iron metabolism scores compared to those in the normal group (**Figure 3B**). To assess the heterogeneity between the two groups, we performed Gene Set Variation Analysis (GSVA), which revealed functional differences between plasma cells with high and low iron metabolism scores (**Figure 3C**). Plasma cells with elevated iron metabolism scores demonstrated enhanced lipogenesis, metabolic activity, and oxidative phosphorylation. Furthermore, we evaluated the expression of antibody secretion-related genes and observed a reduction in antibody secretion functionality in the high iron metabolism group (**Figure 3D**). Cell communication analysis indicated that plasma cells with high iron metabolism scores exhibited stronger communication and signaling output (**Figures 3E, F**). GO enrichment analysis indicated that plasma cells with high iron metabolism scores exhibited increased iron ion transport and oxidative response capabilities (**Figure 3G**). KEGG pathway analysis suggested that these cells were more active in ferroptosis and pyrimidine/nucleotide metabolism (**Figure 3H**). Finally, survival curve analysis revealed that patients with high iron metabolism had shorter overall survival (OS) and progression-free survival (PFS) compared to those with low iron metabolism (**Figures 3I–K**).

### 3.4 Cytotoxic T cell iron metabolism analysis

**Figure 4:**
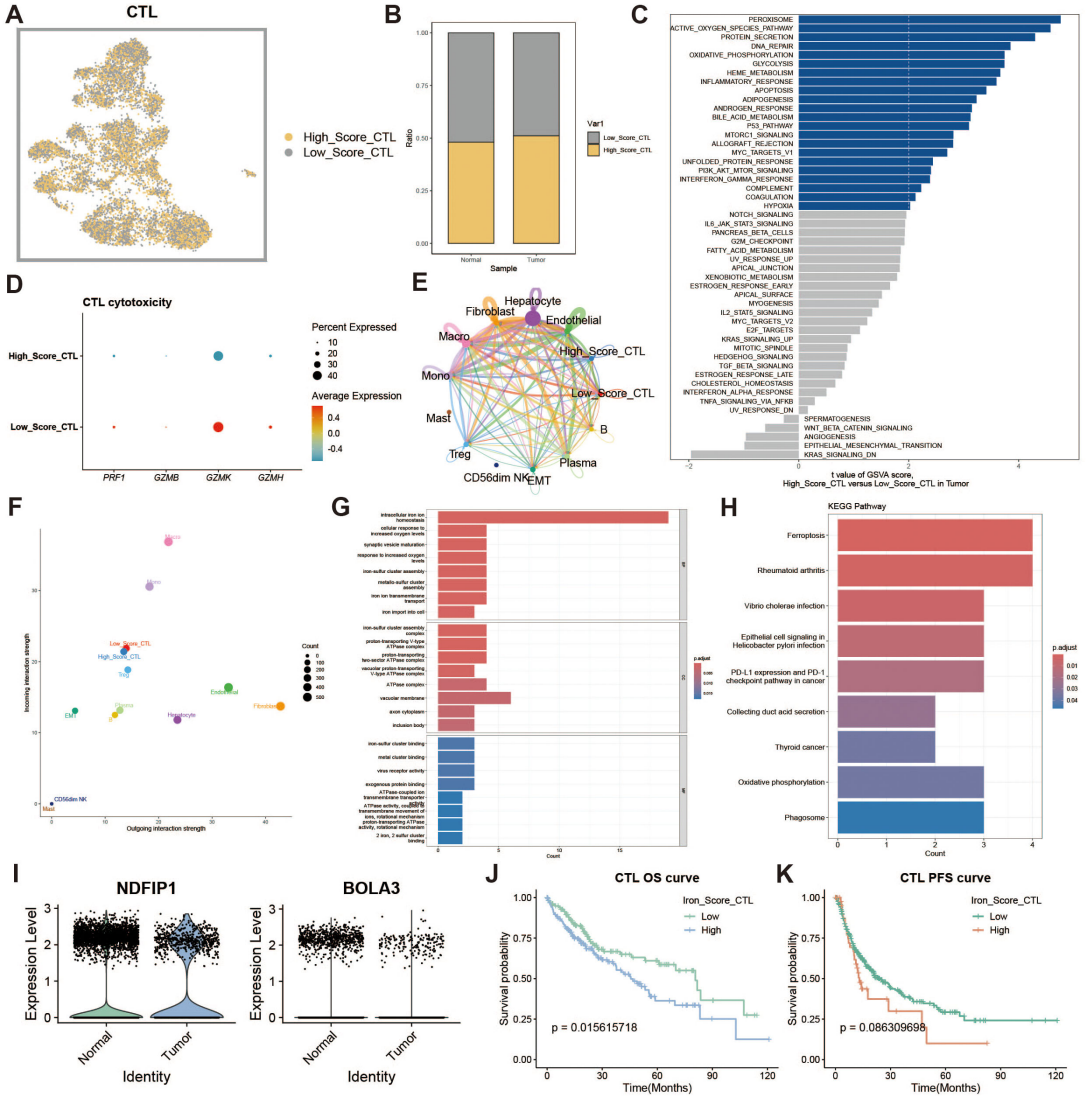
Cytotoxic T cell analysis. **(A)** UMAP of high and low iron metabolism score cells, where yellow indicates high-score cells and gray indicates low-score cells. **(B)** Bar plot of cell proportions. **(C)** Diverging bar plot of GSVA enrichment results for Hallmark gene sets. **(D)** Bubble plot of cytotoxic-related gene expression. **(E)** Circle plot of cell communication frequency. **(F)** Scatter plot of signal transmission and reception strength. **(G)** Bar plot of GO enrichment analysis results. **(H)** Bar plot of KEGG enrichment analysis results. **(I)** Violin plot of iron metabolism-related gene expression in CTLs. **(J)** KM survival curve for overall survival in high and low infiltration groups. **(K)** KM survival curve for progression-free survival in high and low infiltration groups.

To assess the impact of iron metabolism on immune cytotoxicity, we categorized cytotoxic T cells (CTLs) into high and low expression groups based on their iron metabolism scores (**Figure 4A**). Notably, CTLs in the tumor group exhibited significantly higher iron metabolism scores compared to those in the normal group (**Figure 4B**). GSVA analysis of the functional differences between these groups revealed a heightened oxidative profile in the high iron metabolism group (**Figure 4C**). Evaluation of cytotoxicity-related genes showed reduced cytotoxic function in CTLs with elevated iron metabolism (**Figure 4D**). Cell communication analysis further demonstrated enhanced intercellular communication and signal output in these cells (**Figures 4E, F**). GO and KEGG enrichment analyses indicated increased iron ion transport and oxidative responses in CTLs with high iron metabolism (**Figures 4G, H**). Additionally, analysis of iron metabolism-related gene expression revealed a downregulation of NDFIP1 and BOLA3 in tumor tissues (**Figure 4I**). Survival curve analysis showed that patients with high iron metabolism had shorter overall survival (OS) and progression-free survival (PFS) compared to those with low iron metabolism (**Figures 4J, K**).

### 3.5 Effector memory T cell iron metabolism analysis

**Figure 5:**
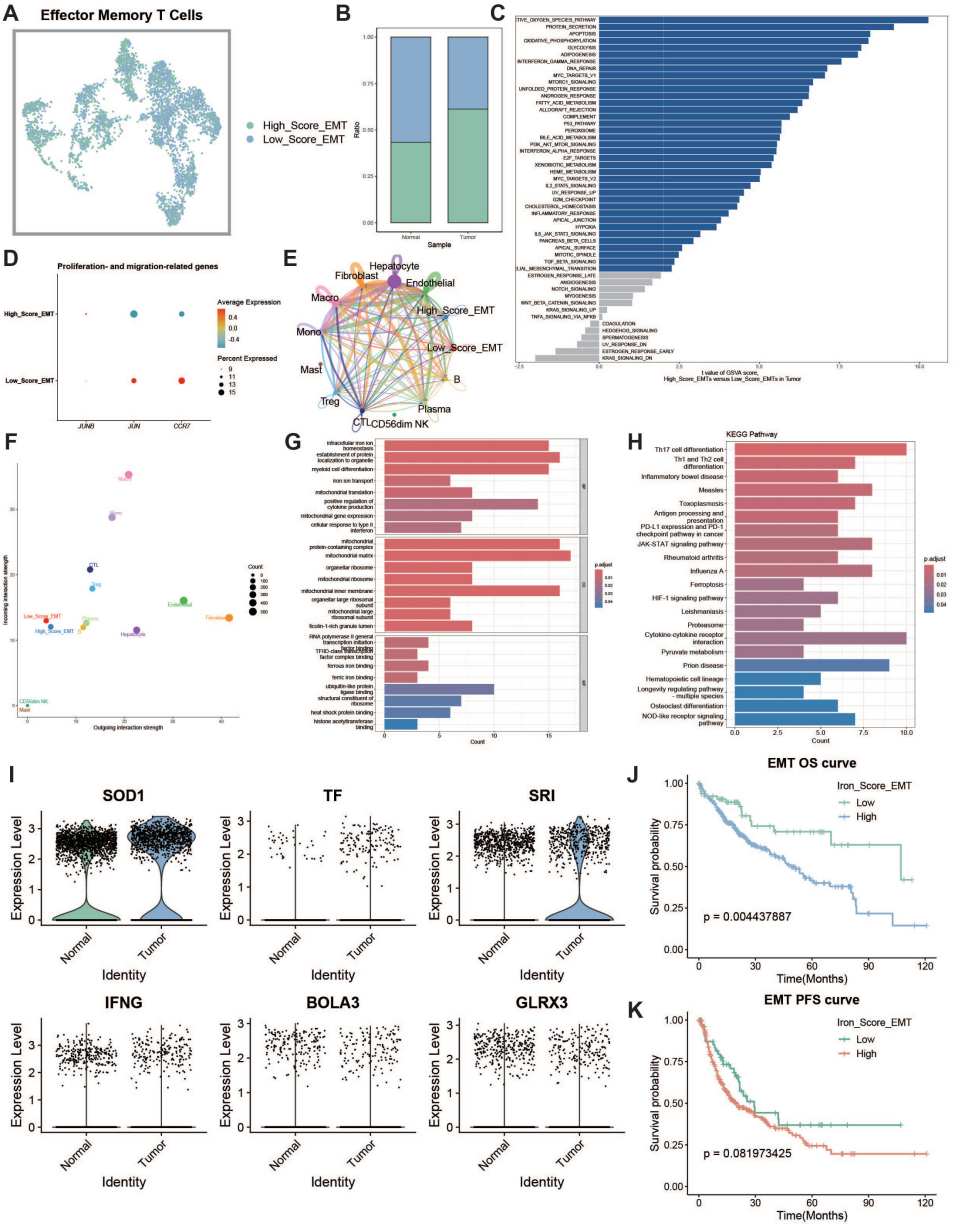
Effector memory T cell analysis. **(A)** UMAP of high and low iron metabolism score cells, with green indicating high-score cells and blue indicating low-score cells. **(B)** Bar plot of cell proportions. **(C)** Diverging bar plot of GSVA enrichment results for Hallmark gene sets. **(D)** Bubble plot of proliferation and migration-related gene expression. **(E)** Circle plot showing cell communication frequency. **(F)** Scatter plot of signal transmission and reception strength. **(G)** Bar plot of GO enrichment analysis results. **(H)** Bar plot of KEGG enrichment analysis results. **(I)** Violin plot of iron metabolism-related gene expression in EMTs. **(J)** KM survival curve for overall survival in high and low infiltration groups. **(K)** KM survival curve for progression-free survival in high and low infiltration groups.

Effector memory T cells (TEMs) can rapidly produce effector cytokines to provide immune protection. We investigated the effects of iron metabolism on their immune function. TEMs were divided into high and low iron metabolism score groups (**Figure 5A**), and TEMs in the tumor group exhibited significantly higher iron metabolism scores than those in the normal group (**Figure 5B**). GSVA analysis revealed increased protein synthesis in high iron metabolism TEMs (**Figure 5C**). However, these cells also exhibited reduced cell proliferation and migration capacities (**Figure 5D**). Cell communication analysis showed enhanced intercellular communication and signal output in TEMs with high iron metabolism (**Figures 5E, F**). GO and KEGG analyses indicated elevated iron ion transport, protein localization, and cell differentiation in high iron metabolism TEMs (**Figures 5G, H**). Additionally, most iron metabolism-related genes were upregulated in tumor tissues (**Figure 5I**). Survival analysis demonstrated shorter overall survival (OS) and progression-free survival (PFS) in patients with high iron metabolism (**Figures 5J, K**).

### 3.6 Regulatory T cell iron metabolism analysis

**Figure 6:**
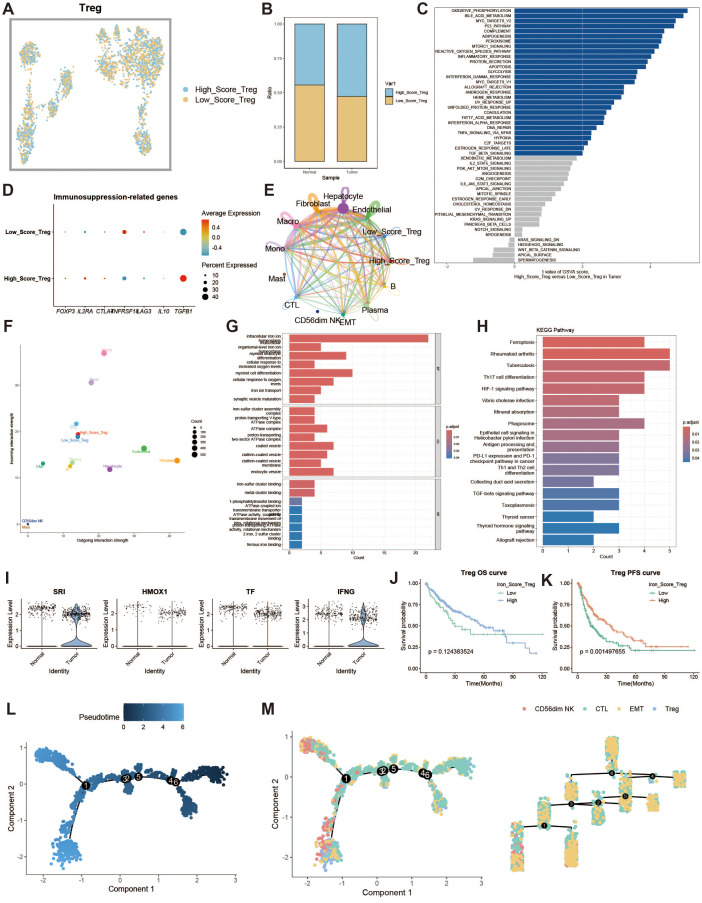
Regulatory T cell analysis. **(A)** UMAP of high and low iron metabolism score cells, with blue indicating high-score cells and yellow indicating low-score cells. **(B)** Bar plot of cell proportions. **(C)** Diverging bar plot of GSVA enrichment results for Hallmark gene sets. **(D)** Bubble plot of immunosuppressive-related gene expression. **(E)** Circle plot of cell communication frequency. **(F)** Scatter plot of signal transmission and reception strength. **(G)** Bar plot of GO enrichment analysis results. **(H)** Bar plot of KEGG enrichment analysis results. **(I)** Violin plot of iron metabolism-related gene expression in Tregs. **(J)** KM survival curve for overall survival in high and low infiltration groups. **(K)** KM survival curve for progression-free survival in high and low infiltration groups. **(L)** Pseudotime trajectory plot of T cell subpopulations, with color representing pseudotime. **(M)** Left panel shows the distribution of T cell subtypes on the trajectory plot, and the right panel shows the trajectory tree diagram.

Regulatory T cells (Tregs) are responsible for modulating immune responses and maintaining self-tolerance. We explored the impact of iron metabolism on their function. Tregs were divided into high and low expression groups based on their iron metabolism scores (**Figure 6A**), and Tregs in the tumor group exhibited higher iron metabolism scores compared to those in the normal group (**Figure 6B**). GSVA analysis revealed that oxidative phosphorylation was a dominant feature in high iron metabolism Tregs (**Figure 6C**). Immune suppression-related genes displayed distinct expression patterns in the high iron metabolism group (**Figure 6D**). Cell communication analysis showed enhanced intercellular communication and signal output in Tregs with high iron metabolism scores (**Figures 6E, F**). GO and KEGG analyses revealed increased iron ion transport and oxidative responses in these cells (**Figures 6G, H**). Interestingly, high iron metabolism was associated with longer overall survival (OS) and progression-free survival (PFS) (**Figures 6I–K**). Pseudotime analysis revealed that CD56dim NK cells and Tregs appeared in the later stages of T cell development (**Figures 6L, M**).

### 3.7 Endothelial cell iron metabolism analysis

**Figure 7:**
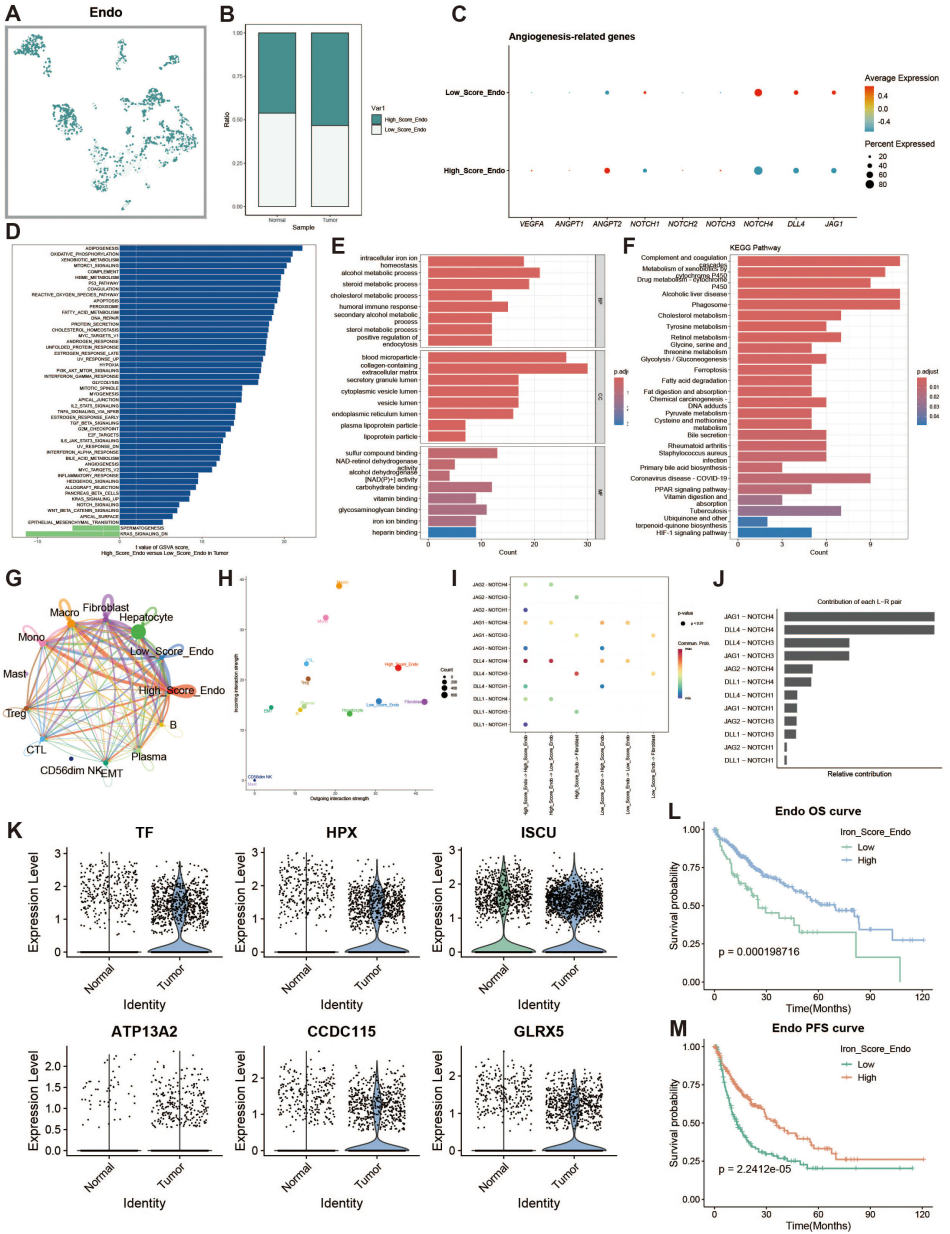
Endothelial cell analysis. **(A)** UMAP of high and low iron metabolism score cells, with green indicating high-score cells and white indicating low-score cells. **(B)** Bar plot of cell proportions. **(C)** Bubble plot of angiogenesis-related gene expression. **(D)** Diverging bar plot of GSVA enrichment results for Hallmark gene sets. **(E)** Bar plot of GO enrichment analysis results. **(F)** Bar plot of KEGG enrichment analysis results. **(G)** Circle plot of cell communication frequency. **(H)** Scatter plot of signal transmission and reception strength. **(I)** Bubble plot showing NOTCH signaling pathway communication between high and low iron metabolism endothelial cells. **(J)** Contribution of ligand-receptor pairs to communication in the NOTCH pathway. **(K)** Violin plot of iron metabolism-related gene expression in endothelial cells. **(J)** KM survival curve for overall survival in high and low infiltration groups. **(K)** KM survival curve for progression-free survival in high and low infiltration groups.

To explore iron metabolism in endothelial cells within the liver cancer microenvironment, we divided endothelial cells into high and low expression groups based on iron metabolism scores (**Figure 7A**). Endothelial cells in the tumor group exhibited higher iron metabolism scores compared to those in the normal group (**Figure 7B**). Endothelial cells with high iron metabolism showed upregulation of pro-angiogenic genes, such as VEGFA (**Figure 7C**). GSVA analysis indicated increased oxidative phosphorylation and metabolic activity in high iron metabolism endothelial cells (**Figure 7D**). GO and KEGG enrichment analyses revealed elevated metabolic activity in these cells (**Figures 7E, F**). Cell communication analysis showed enhanced intercellular communication and signal output (**Figures 7G, H**). The JAG1-NOTCH1 pathway was identified as a key mediator of active communication (**Figures 7I, J**). Survival analysis revealed that patients with high iron metabolism had a longer survival period (**Figures 7L, M**).

### 3.8 Characterization of iron metabolism in fibroblasts

**Figure 8:**
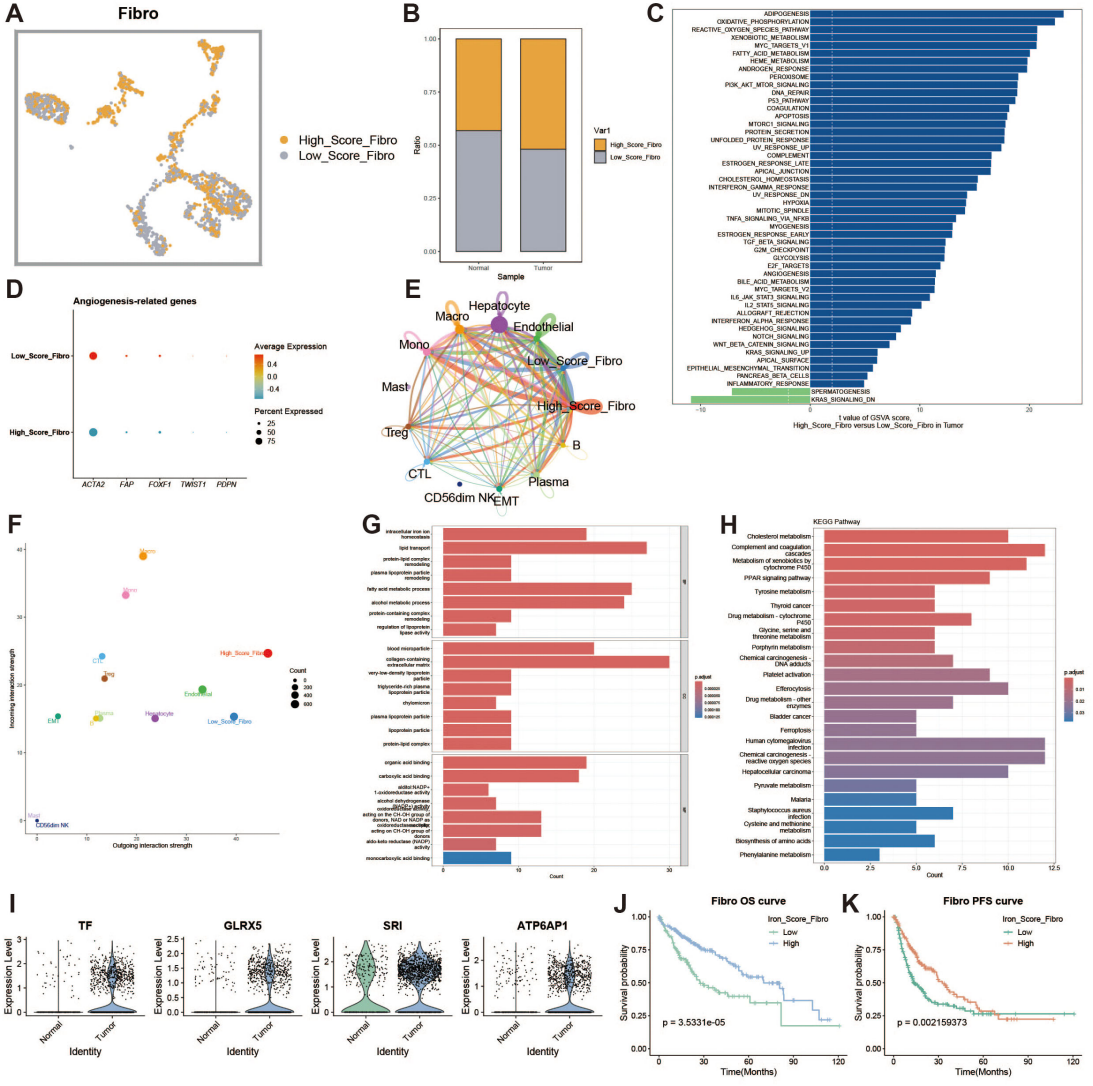
Fibroblast analysis. **(A)** UMAP of high and low iron metabolism score cells, with yellow indicating high-score cells and gray indicating low-score cells. **(B)** Bar plot of cell proportions. **(C)** Diverging bar plot of GSVA enrichment results for Hallmark gene sets. **(D)** Bubble plot of tumor-associated fibroblast marker gene expression. **(E)** Circle plot of cell communication frequency. **(F)** Scatter plot of signal transmission and reception strength. **(G)** Bar plot of GO enrichment analysis results. **(H)** Bar plot of KEGG enrichment analysis results. **(I)** Violin plot of iron metabolism-related gene expression in fibroblasts. **(J)** KM survival curve for overall survival in high and low infiltration groups. **(K)** KM survival curve for progression-free survival in high and low infiltration groups.

Fibroblasts are an important cellular component of the tumor microenvironment. Tumor-associated fibroblasts play a key role at all stages of tumor development, promoting tumor proliferation and migration, enhancing tumor angiogenesis, regulating tumor immunity, and increasing tumor drug resistance. We divided fibroblasts into high- and low-expression groups based on iron metabolism scores (**Figures 8A, B**). To explore the heterogeneity between the two groups, we analyzed the functional differences between fibroblasts with high and low iron metabolism scores through GSVA and found that the high iron metabolism score group exhibited higher oxidative phosphorylation, reactive oxygen species response, and fat generation-related characteristics (**Figure 8C**). We constructed a bubble chart to visualize the expression levels of tumor-associated fibroblast marker genes, aiming to explore the impact of different metabolic scores on the generation of tumor-associated fibroblasts (**Figure 8D**). The results indicated that the expression of tumor-associated fibroblast marker genes was elevated in fibroblasts with low iron metabolism scores. In a cell communication analysis, we discovered that fibroblasts with high iron metabolism scores exhibited enhanced levels of cell communication and stronger signal output characteristics (**Figures 8E, F**). Gene Ontology (GO) enrichment analysis revealed that fibroblasts with high iron metabolism scores demonstrated increased iron ion transport characteristics and a heightened oxidative response (**Figure 8G**).KEGG pathway analysis found that fibroblasts with high iron metabolism scores displayed more active metabolism (**Figure 8H**). We further analyzed the expression levels of iron metabolism genes and found that most of these genes were downregulated in liver cancer tissues (**Figure 8I**). Survival curves indicated that patients with high iron metabolism had a longer survival period compared to those with low iron metabolism (**Figures 8J, K**).

### 3.9 Spatial distribution of iron metabolism

**Figure 9:**
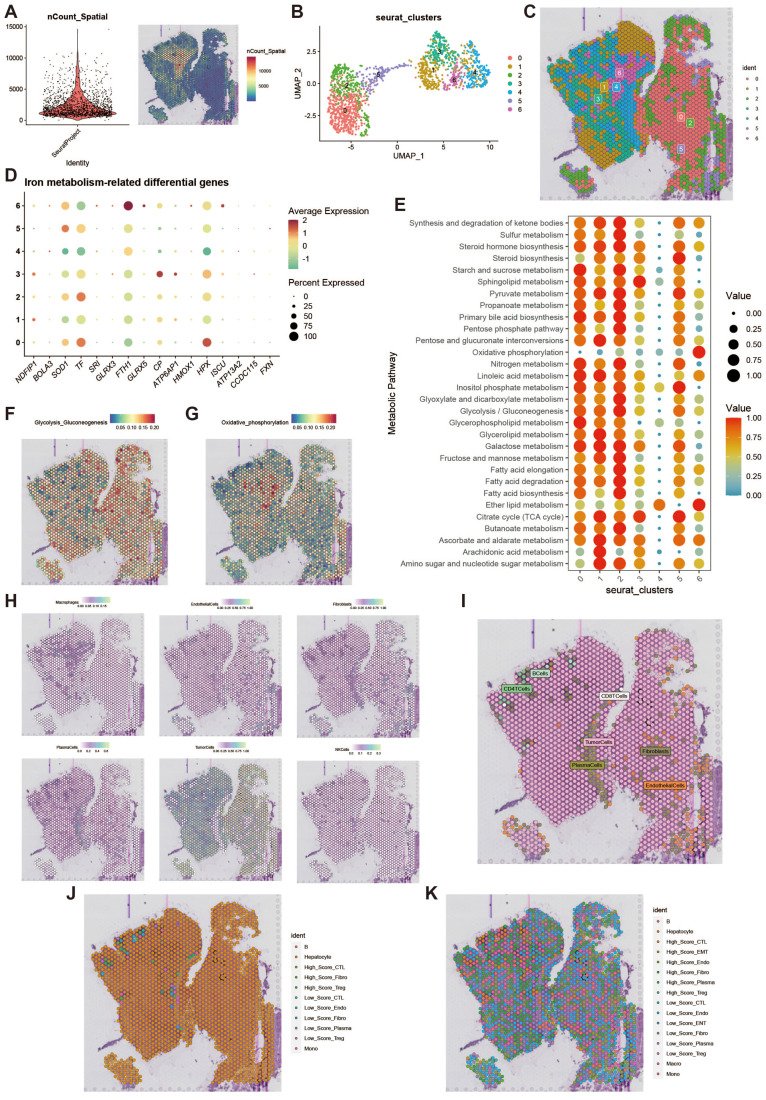
Spatial transcriptomics deconvolution analysis. **(A)** Heatmap of count values on spatial transcriptomic slices. **(B)** UMAP of dimensionality-reduced clustering results. **(C)** Plot of reduced dimensional clustering on spatial transcriptomic slices. **(D)** Bubble plot showing expression of key iron metabolism-related genes in spatial transcriptomics data. **(E)** Bubble plot of metabolic pathway activity scores. **(F)** Heatmap of glycolysis activity. **(G)** Heatmap of oxidative phosphorylation activity. **(H)** Deconvolution analysis results, including tumor cells, macrophages, fibroblasts, etc. **(I)** Plot of the most likely cell type for each spot. **(J)** Deconvolution analysis incorporating high and low iron metabolism levels, showing primary cell type results. **(K)** Deconvolution analysis incorporating high and low iron metabolism levels, showing secondary cell type results.

To further investigate the features of iron metabolism in liver cancer, we conducted a deconvolution analysis of spatial transcriptomic data. We obtained spatial transcriptomic sequencing data from hepatocellular carcinoma (HCC) tumor tissue (GSM6177612), specifically from the tumor region of primary hepatocellular carcinoma. Following dimensionality reduction and clustering of the spatial transcriptomic data, we visualized the results using UMAP, which revealed seven distinct cell clusters (**Figures 9A, B**). The spatial distribution of these cell clusters is illustrated in **Figure 9C**. We assessed the iron metabolism-related gene scores for each cell cluster (**Figure 9D**) and analyzed the metabolic differences among the clusters, highlighting elevated metabolic activity in clusters 0, 1, and 2 (**Figure 9E**). Additionally, we examined the activity levels of glycolytic and oxidative phosphorylation metabolic pathways across different spatial regions (**Figures 9F, G**). High metabolic activity generally indicates that these cells play a more active role in tumor growth and progression, particularly in scenarios where energy demands are elevated. The spatial differences in the glycolytic and oxidative phosphorylation pathways suggest that cells in different regions may employ unique metabolic strategies to adapt to changes in the microenvironment. To further clarify the metabolic characteristics of cells in each spot and reveal the spatial distribution of their iron metabolism levels, we displayed the single-cell annotation results at the spatial level through deconvolution analysis (**Figures 9H, I**), showing the primary and secondary probabilities of cells with different iron metabolism levels in each spot (**Figures 9J, K**).

### 3.10 Survival analysis of iron metabolism-related genes

**Figure 10:**
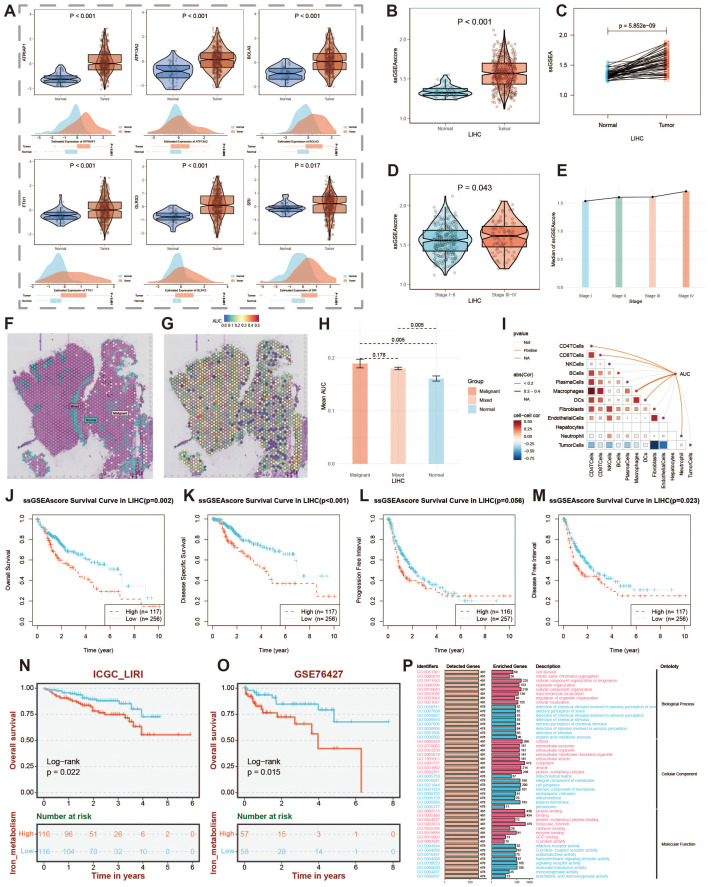
Expression of key iron metabolism-related genes and prognosis analysis. **(A)** Violin plot showing differential expression of key iron metabolism-related genes in TCGA and GEO data. **(B)** Violin plot of ssGSEA score results. **(C)** Differential expression of ssGSEA scores in paired samples. **(D)** Violin plot of score differences among patients at different clinical stages. **(E)** Line chart showing changes in scores across different clinical stages. **(F)** Identification of malignant, mixed, and normal cells in spatial transcriptomics data. **(G)** Active landscape of key iron metabolism-related genes in microregions. **(H)** Differences in the AUC scores of key iron metabolism-related genes between malignant, mixed malignant, and normal microregions at spatial transcriptomics resolution. **(I)** Spearman correlation between the AUC scores of key iron metabolism genes and microenvironment components. **(J-M)** Prognostic curves of patients with high and low iron metabolism scores. **(N)** Prognostic curve in ICGC-LIRI data. **(O)** Prognostic curve in GSE76427 data. **(P)** GO enrichment analysis of patients with high and low iron metabolism scores.

We analyzed the differential expression of key iron metabolism genes in tumor versus normal samples using TCGA and GEO data (**Figure 10A**), and performed ssGSEA scoring of iron metabolism levels in both groups (**Figures 10B, C**). The results revealed that iron metabolism levels were markedly elevated in the tumor group compared to the normal group. To further investigate the role of iron metabolism across different tumor stages, we examined and illustrated the differences in iron metabolism scores among patients at various clinical stages (**Figures 10D, E**). In the spatial transcriptomics data, we identified malignant, mixed, and normal cells, and depicted the iron metabolism score intensities for these three cell types (**Figures 10F–H**). The elevated iron metabolism scores in malignant cells compared to normal cells reflect the heterogeneity of iron metabolism within the tumor microenvironment. The correlation between AUC scores of key iron metabolism genes and microenvironment components further indicated that iron metabolism plays a significant role in regulating the tumor microenvironment, potentially influencing intercellular metabolic communication and tumor growth.

Through Spearman correlation analysis of AUC scores for key iron metabolism genes and microenvironment components, we further validated the pivotal role of iron metabolism in liver cancer (**Figure 10I**). We extracted overall survival (OS), disease-free survival (DFS), progression-free interval (PFI), and disease-free interval (DFI) data from liver cancer samples and examined the survival durations of patients with varying iron metabolism levels. The results indicated that higher iron metabolism levels were associated with poorer prognoses across these survival metrics (**Figures 10J–M**). This suggests that elevated iron metabolism levels may serve as a potential biomarker for increased tumor malignancy and adverse prognosis. Additionally, we utilized ICGC-LIRI and GSE76427 datasets to generate prognostic curves, thereby corroborating our findings (**Figures 10N, O**). Furthermore, conducting Gene Ontology (GO) enrichment analysis on patients with high and low iron metabolism scores revealed potential mechanisms through which iron metabolism influences prognosis (**Figure 10P**). Overall, these results suggest that iron metabolism represents a critical target for diagnosis and treatment in liver cancer.

### 3.11 GLRX3 expression and prognostic analysis

**Figure 11:**
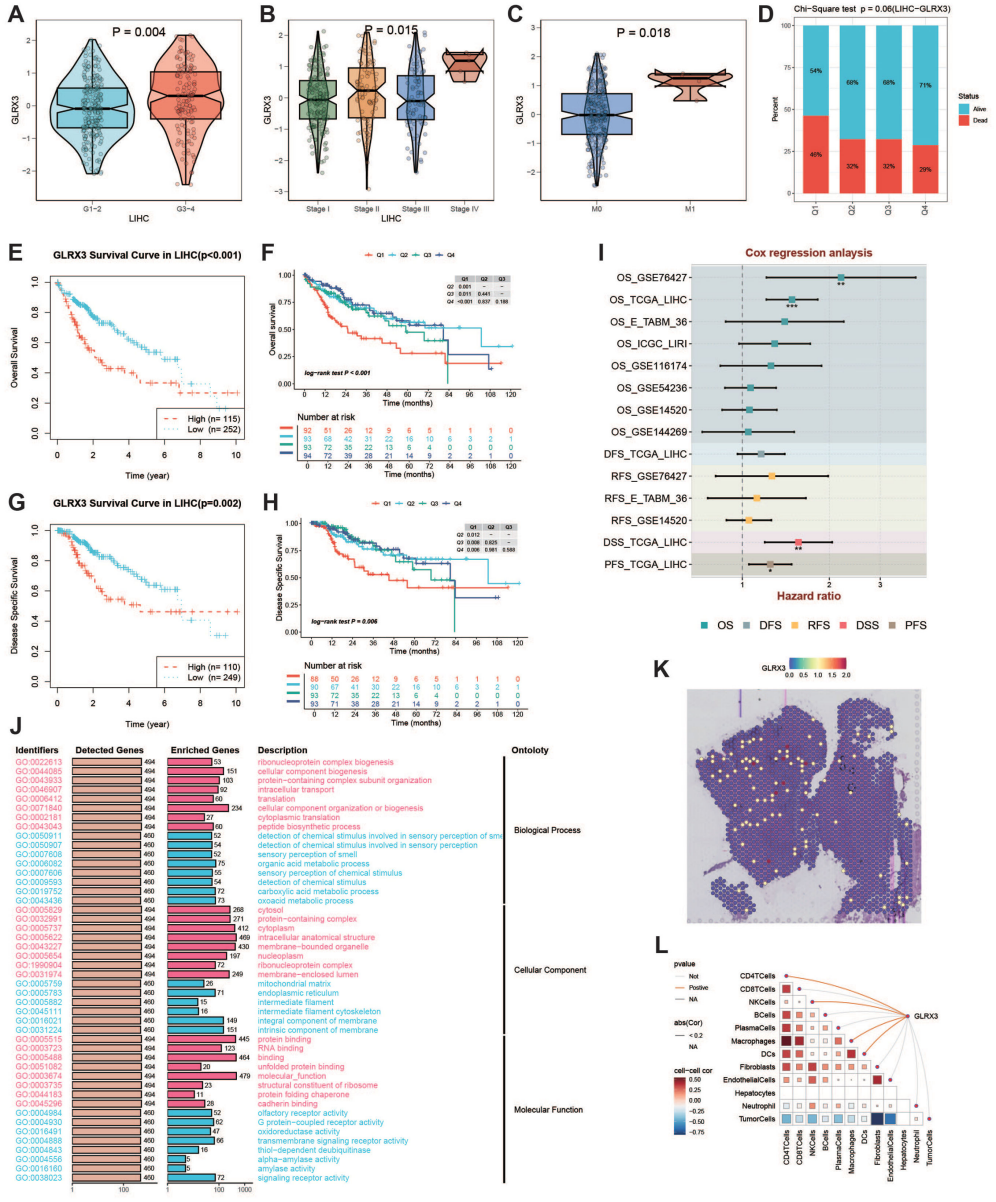
Clinical subgroup expression and K-M curve of GLRX3. **(A)** Differential expression of GLRX3 in high/low tumor grades in the TCGA cohort. **(B)** Differential expression of GLRX3 in high and low stages in the TCGA cohort. **(C)** Differential expression of GLRX3 in M1 and M0 stages in the TCGA cohort. **(D)** Bar chart of chi-square test showing the number of survival and death samples with different expression levels. The X-axis represents patients with different GLRX3 expression levels, and the Y-axis represents the proportion of deaths (red) and survivors (blue). **(E)** Kaplan-Meier survival analysis of OS. **(F)** Kaplan-Meier survival analysis of OS dividing patients into four groups (Q1, Q2, Q3, and Q4) based on GLRX3 expression levels. **(G)** Kaplan-Meier survival analysis of DSS. **(H)** Kaplan-Meier survival analysis of DSS dividing patients into four groups (Q1, Q2, Q3, and Q4) based on GLRX3 expression levels. **(I)** Meta-analysis of survival risk ratios. **(J)** GO enrichment analysis of high and low expression groups. **(K)** Each dot represents a microregion (spot) from spatial transcriptomics sequencing. The darker the color (red), the higher the expression level of the gene in the spot. **(L)** Correlation between cell content and GLRX3 expression levels in all spots, and correlation between cell content and GLRX3 gene expression.

GLRX3, a key iron metabolism gene, was found to be highly expressed in HCC. Glutaredoxin 3 (GLRX3) is a type II monothiol glutaredoxin involved in iron balance, redox reactions, and antioxidant responses. In the TCGA cohort, GLRX3 expression was higher in advanced tumor grades (**Figure 11A**) and higher-stage tumors (**Figure 11B**). M1-stage tumors also showed increased GLRX3 expression compared to M0-stage tumors (**Figure 11C**). These differences indicate that high GLRX3 expression correlates with more advanced tumors and poorer prognosis (**Figure 11D**). We validated these findings by analyzing OS and DSS in patients with different GLRX3 expression levels (**Figures 11E–H**). Meta-analysis confirmed our conclusions (**Figure 11I**). GO enrichment analysis of high and low GLRX3 score patients revealed potential mechanisms influencing prognosis (**Figure 11J**). Spatial transcriptomics data showed that GLRX3 was highly expressed in malignant regions (**Figure 11K**). Spearman correlation analysis further confirmed the role of GLRX3 in HCC (**Figure 11L**).

### 3.12 Knocking down the expression level of GLRX3 significantly inhibited the proliferation, invasion and migration of hepatocellular carcinoma cells

**Figure 12:**
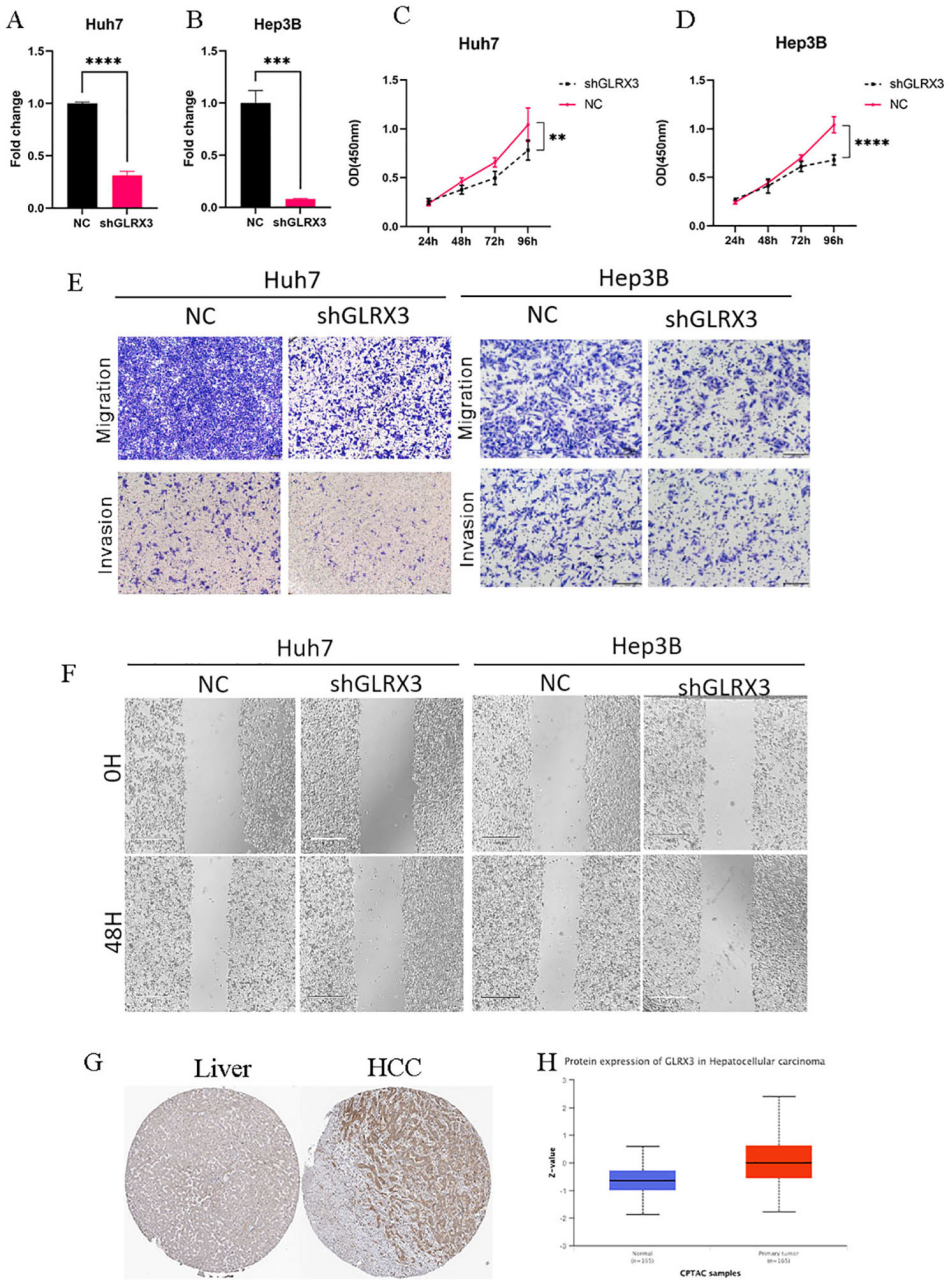
*In vitro* experiments to validate the role of GLRX3 in hepatocellular carcinoma. **(A, B)** PCR assay to detect GLRX3 knockdown efficiency. **(C, D)** CCK8 assay to detect cell viability. **(E)** transwell assay. **(F)** Wound healing assay. **(G)** IHC assay. **(H)** CTPAC database to verify GLRX3 expression. ** represents a p-value < 0.01, *** represents a p-value < 0.001, **** represents a p-value < 0.0001.

Knocking down the expression level of GLRX3 significantly inhibited the proliferation, invasion, and migration of hepatocellular carcinoma cells. Considering the importance of GLRX3, we validated its role in hepatocellular carcinoma through a series of *in vitro* experiments. First, we reduced the expression of GLRX3 and confirmed via PCR that its level was significantly decreased compared to the control group (**Figures 12A, B**). Subsequently, CCK8 assays demonstrated that the knockdown of GLRX3 markedly inhibited the activity of hepatocellular carcinoma cells (**Figure 12C, D**). To investigate the relationship between GLRX3 and the invasive migration of hepatocellular carcinoma, we conducted transwell and wound healing assays, revealing that GLRX3 knockdown significantly inhibited the invasive migration of these cells (**Figures 12E, F**). Immunohistochemistry experiments indicated that GLRX3 was highly expressed in hepatocellular carcinoma tissues (**Figure 12G**), and Western blot analysis confirmed the elevated protein expression of GLRX3 in these tissues (**Figure 12H**). In summary, GLRX3 enhances the invasive migration of hepatocellular carcinoma cells, correlating with the malignant characteristics of the disease.

## 4 Discussion

Iron serves dual roles in cancer biology: it acts as an initiator in the early stages of tumor development and functions as a promoter during malignancy, allowing transformed cells to maximize their potential for uncontrolled proliferation. Concurrently, cancer cells exhibit an increased demand for iron to support cellular growth, leading to alterations in iron metabolism-related gene expression that facilitate enhanced iron acquisition. Previous studies have demonstrated that tumor cells often upregulate transferrin receptor 1 (TFR1) while downregulating ferroportin (FNP), thereby limiting iron release (4, 19, 21). In our study, by scoring iron metabolism-related genes, we classified all cells into high and low iron metabolism groups and found that iron metabolism activity was consistently higher in tumor samples.

Disruption of the cellular iron homeostasis mechanism can lead to abnormal iron accumulation or depletion within cells. Under normal conditions, cells finely regulate iron levels to maintain a balance between demand and supply (39, 40). Heightened iron metabolism can disrupt homeostasis, resulting in abnormal iron levels that adversely impact cellular function and overall health. This dysregulation may alter the cellular redox balance, potentially inducing oxidative damage and ultimately resulting in iron-dependent programmed cell death, known as ferroptosis. These factors can significantly impact the prognosis of liver cancer patients (41). In our study, we generated multiple prognostic curves based on iron metabolism, showing that patients with higher iron metabolism scores experienced worse outcomes to varying degrees (42). Similarly, elevated iron metabolism was correlated with advanced tumor grades and stages. These findings underscore the potential of abnormal iron metabolism as a predictive biomarker and therapeutic target in cancer. They provide single-cell-level evidence to support the clinical investigation of iron chelators in cancer therapy (43). For example, oral iron chelators, such as deferasirox, have shown efficacy in leukemia patients, while the thiosemicarbazone Dp44mT has inhibited cancer cell proliferation *in vitro* by inducing the expression of p21, a cyclin-dependent kinase inhibitor involved in cell cycle arrest (44–46).

Our study also identified GLRX3 (Glutaredoxin 3) as a key iron metabolism target gene that significantly influences liver cancer progression (47). GLRX3 is a critical iron-sulfur cluster protein primarily involved in regulating iron metabolism. As a member of the oxidoreductase family, it performs multiple biological roles within cells, particularly in maintaining iron homeostasis and facilitating the assembly and transport of iron-sulfur clusters. In our study, we observed that GLRX3 was abnormally expressed in liver cancer patients (48). Iron-sulfur clusters serve as essential cofactors for many enzymes and proteins. Overexpression of GLRX3 can enhance the assembly and transport of these clusters in the cytoplasm, leading to excessive production and distribution (49). This overactivation may disrupt the metabolic balance in certain cells by over activating iron-sulfur cluster-dependent proteins. This finding is consistent with previous studies showing that tumor cells increase their demand for iron to sustain proliferation, with alterations in iron metabolism gene expression facilitating iron acquisition (50). Consequently, the expression level of GLRX3 may serve as a significant biomarker for liver cancer prognosis and as a potential indicator for assessing the efficacy of immunotherapy. Future research should prioritize elucidating the specific mechanisms through which GLRX3 contributes to tumor progression and developing targeted therapeutic strategies to enhance prognosis and treatment outcomes for liver cancer patients (51).

Although our study reveals the critical impact of iron metabolism on liver cancer, several limitations should be noted. First, the limited sample size may affect the generalizability of our findings. We hope future studies will analyze larger datasets, incorporating single-cell data from liver cancer patients across different databases, to fully explore the effects of iron metabolism dysregulation on the tumor microenvironment. Second, future studies should integrate proteomics and metabolomics approaches to provide multi-omics analyses that better elucidate the functional role of GLRX3. Third, due to technical and funding constraints, while we investigated the impact of GLRX3 on liver cancer prognosis, we did not conduct a comprehensive analysis of other key iron metabolism genes. Future research should broaden the scope of iron metabolism studies in liver cancer (4). Despite these limitations, our understanding of many processes remains incomplete, but growing recognition of the importance of iron metabolism in cancer biology offers new opportunities to uncover the mechanisms driving tumorigenesis. This, in turn, could lead to the development of more effective iron-targeted therapies for liver cancer.

## 5 Conclusion

This study revealed that iron metabolism plays a critical role in the progression of liver cancer, focusing on the role of GLRX3 (Glutaredoxin 3) in modulating iron homeostasis and driving tumor progression. The study showed that disruptions in iron metabolism lead to abnormal iron accumulation or deficiency within liver cancer cells, inducing oxidative damage and ferroptosis. GLRX3, a key regulatory protein for iron-sulfur clusters, is abnormally overexpressed in liver cancer patients, and its overexpression facilitates iron-sulfur cluster assembly and transport, thereby disrupting metabolic balance and promoting tumor cell growth and metastasis. Survival analysis and experimental validation demonstrated that high GLRX3 expression correlates with poor patient prognosis, highlighting its potential as a prognostic biomarker and an indicator for assessing immune therapy response.

## Data Availability

The original contributions presented in the study are included in the article/supplementary material, further inquiries can be directed to the corresponding author/s.
